# Methodological Considerations for Using Hair Cortisol as a Biomarker Following No Measurable Effects of Tactile Massage in Adolescents With ADHD

**DOI:** 10.1155/ijpe/5891199

**Published:** 2026-10-21

**Authors:** Carl-Johan Törnhage, Anna-Carin Robertz, Anne-Katrin Kantzer, Stefan Nilsson

**Affiliations:** ^1^ Sahlgrenska Academy Institution for Clinical Science University of Gothenburg Gothenburg Sweden gu.se; ^2^ Department of Paediatrics Skaraborg Hospital Skövde Sweden; ^3^ Department of Research and Development Skaraborg-Hospital Group Skövde Sweden; ^4^ Institute of Health and Care Sciences Sahlgrenska Academy University of Gothenburg Gothenburg Sweden gu.se; ^5^ Child and Adolescent Psychiatry Clinic NU Hospital Group Trollhättan Sweden; ^6^ Centre for Person-Centred Care (GPCC) Sahlgrenska Academy University of Gothenburg Gothenburg Sweden gu.se; ^7^ Queen Silvia Children′s Hospital Sahlgrenska University Hospital Gothenburg Sweden sahlgrenska.se

**Keywords:** ADHD, adolescents, cortisol, hair, tactile massage

## Abstract

**Aims:**

Dysregulation of the hypothalamic–pituitary–adrenal axis has been proposed as a mechanism in attention‐deficit/hyperactivity disorder (ADHD). Tactile massage is suggested to influence long‐term cortisol secretion in adolescents with ADHD, although it remains unclear whether such an effect occurs. This study examined whether tactile massage alters hair cortisol in adolescents with ADHD.

**Methods:**

Fourteen adolescents aged 15–17 with ADHD received 10 weekly 60‐min standardised tactile massage sessions. Hair samples were collected at five time points and analysed for cortisol using a competitive radioimmunoassay. Perceived stress (the Stress in Children questionnaire), heart rate and blood pressure were assessed. Non‐parametric statistics (Friedman′s ANOVA, Wilcoxon matched‐pairs tests and Spearman′s correlation) evaluated time‐dependent changes and associations.

**Results:**

Hair cortisol showed no significant differences between baseline, intervention and follow‐up (*p* = 0.63). After six sessions, boys had higher cortisol levels than girls (*p* = 0.029). No significant correlations emerged between hair cortisol and heart rate, blood pressure or total stress, although a negative correlation was found with frustration (*p* = 0.028).

**Conclusions:**

Because the intervention did not alter long‐term cortisol output, and hair cortisol shows limited sensitivity to short‐term effects, it does not appear suitable for detecting tactile‐massage effects in adolescents with ADHD.

Summary

Consensus is lacking as to whether hair cortisol is an appropriate biomarker for detecting the effects of tactile massage on adolescents with ADHD or whether tactile massage produces sustained cortisol changes. The present study found no significant changes in the adolescents′ hair cortisol concentrations after 10 massage sessions. Consequently, hair cortisol does not appear to be a suitable measure for detecting the effects of tactile massage in adolescents with ADHD.

## 1. Introduction

The neurobiological mechanisms underlying attention‐deficit/hyperactivity disorder (ADHD) are not fully understood, but dysregulation of the hypothalamic–pituitary–adrenal (HPA) axis has been proposed as a central pathophysiological mechanism [1–3]. Cortisol, the primary end product of the HPA axis, is widely used as a peripheral biomarker of stress‐system activity [4, 5]. In adolescents with ADHD, cortisol findings are particularly heterogeneous and appear strongly influenced by psychiatric comorbidity, sex and recruitment strategy. Studies measuring cortisol in saliva or blood have frequently reported lower basal cortisol levels and blunted stress responses in adolescents with ADHD compared with healthy controls [3, 6]. However, findings remain heterogeneous, with some studies reporting no differences or even elevated cortisol levels in adolescents with ADHD [2, 6]. These inconsistencies are partly attributable to methodological limitations, as traditional cortisol measures capture the total amount of cortisol in blood and the free biologically active cortisol in saliva, which reflect short‐term fluctuations and are strongly influenced by circadian rhythms and acute stressors [2, 5, 7].

Hair cortisol concentration has been widely applied across age groups, clinical conditions and intervention studies to assess chronic stress and long‐term cortisol output [5, 8–14]. Developmental studies of hair cortisol concentration in children and adolescents show mixed findings. Some report decreasing hair cortisol concentration with increasing age and no sex differences [8], whereas others observe increasing hair cortisol concentration with age, or a correlation with sex, as well as with bodily stress because of poor health [15, 16].

Sex‐specific associations have also been described, with low hair cortisol concentration being more strongly related to inattention in boys with ADHD. For this group, positive associations between hair cortisol concentration and cognitive performance in attention and memory domains have also been observed [1, 2]. Differences in symptom severity, medication exposure and dimensional versus categorical assessment approaches may further contribute to inconsistent findings [6].

Tactile massage is a gentle, structured form of skin stimulation using slow, rhythmic strokes that activate tactile receptors, specifically C‐tactile fibres, which respond optimally to touch at approximately 1–10 cm/s [17, 18]. Activation of these fibres has been associated with stress reduction, pain relief and improved well‐being [19]. However, no evidence‐based studies have evaluated tactile massage in children and adolescents with ADHD.

The present study addresses this gap by analysing hair cortisol concentration in adolescents with ADHD participating in a tactile massage intervention study [20], and examining associations with heart rate, blood pressure and self‐perceived stress (the Stress in Children [SIC] questionnaire) [21].

### 1.1. Aims and Hypothesis

This study is aimed at assessing changes in hair cortisol at baseline, during the tactile massage intervention and at follow‐up.

The hypothesis was that hair cortisol concentration would decrease during the tactile massage intervention and subsequently increase following its termination.

We also hypothesised that hair cortisol concentration would be an appropriate measure for detecting sustained changes in long‐term HPA‐axis activity associated with repeated tactile massage.

## 2. Patients and Methods

### 2.1. Study Design

Data collection occurred at five time points (T0–T4) and included blood pressure, heart rate, hair cortisol and the SIC. The inclusion and exclusion criteria and the data collection process are illustrated in Figure 1.

**Figure 1:**
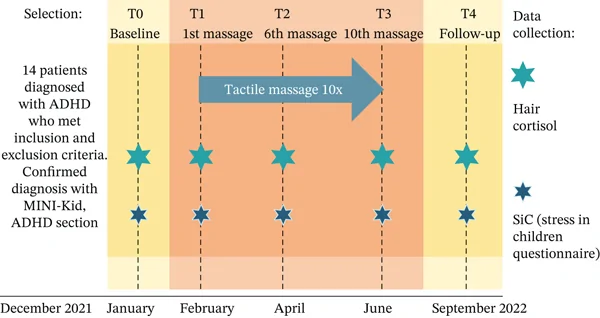
The inclusion and exclusion criteria and the data collection process.

### 2.2. Study Population

Inclusion criteria were adolescents: (1) aged 15–17 years; (2) previously diagnosed with ADHD in the combined form F90.0B according to the International Classification of Diseases (ICD‐10) (WHO, 2004); (3) unmedicated or medicated with central stimulants and/or atomoxetine without modifications for the previous 6 months. Unmedicated or medicated with sleep medication, such as melatonin and/or promethazine; (4) Ability to speak, write and read Swedish.

Exclusion criteria were: (1) ongoing suicidality; (2) ongoing substance abuse; (3) other severe mental disorders such as autism, severe depression, epilepsy, manic episode or psychosis and intellectual disability; (4) engagement/involvement in other ongoing psychological treatment such as psychotherapy, group therapy and parent training.

Recruitment followed a predetermined structure and concluded once 14 adolescents had consented to participate. The sample included nine boys (64%); four participants were 15 years old, seven were 16 and three were 17. All adolescents provided hair samples. Baseline characteristics (T0) are presented in Table 1. *p* − level < 0.05 was considered significant.

**Table 1:** Characteristics of participants at baseline given as mean and SD.

	Female	Male	All	*p* ^e^
	(*N* = 5)	(*N* = 9)	(*N* = 14)	
Age	16.1 (0.5)	16.5 (0.7)	16.4 (0.4)	Ns
Weight	56.6 (13.2)	69.9 (13.0)	65.1 (1.4)	Ns
Height	164.0 (10.5)	180.0 (4.4)	174.3 (6.4)	0.004
BMI^g^	20.9 (3.8)	21.6 (3.9)	21.3 (3.7)	Ns
Pharmacy				
Adhd^a^	4	7	11	Ns
Sleep^b^	1	3	4	Ns^f^
SSRI^c^	1	0	1	Ns^f^
Heart rate	86 (11)	75 (13)	78 (10)	0.029
Systolic BP^d^	106 (9)	120 (10)	115 (1)	Ns
Diastolic BP^d^	73 (8)	74 (7)	73 (8)	Ns
SIC (cut off 2.5)				
Well‐being	2.1 (0.6)	2.3 (0.5)	2.3 (0.5)	Ns
Frustration	2.4 (0.5)	2.1 (0.4)	2.2 (0.7)	Ns
Social support	2.0 (0.4)	1.8 (0.4)	1.9 (0.1)	Ns
Total score	2.2 (0.4)	2.0 (0.3)	2.1 (0.4)	Ns

Abbreviations: Ns, nonsignificant; ODD, oppositional defiant disorder.

^a^Lisdexamphetamine, dexamphetamine, methylphenidate and atomoxetine.

^b^Melatonin and promethazine.

^c^Sertraline.

^d^BP (blood pressure (mmHg).

^e^MW‐U comparing sex.

^f^Fisher′s exact test.

^g^BMI reference values for female: mean (SD) 15 yrs 19.8 (2.5), 16 yrs 20.4 (1.9) and 17 yrs 21.0 (2.3), and for male: mean (SD) 15 yrs 19.6 (2.2), 16 yrs 20.3(2.3) and 17 yrs 21.1(2.3).

### 2.3. Concomitant Medication Use Across the Study Period

#### 2.3.1. ADHD Medication

Of the 14 participants, 11 were treated with ADHD medication, generally within the therapeutic range. Five participants had medication changes during the intervention: two discontinued ADHD medication by T4, one increased methylphenidate dosage, one switched from a therapeutic dose of methylphenidate to a starting dose of Lis‐dexamphetamine and one initiated ADHD medication between T0 and T1.

#### 2.3.2. Sleep Medication

Four participants received medication for sleep problems, with no changes during the study.

#### 2.3.3. Other Medication

One participant began selective serotonin reuptake inhibitor treatment for anxiety and depression after T3, and one received esomeprazole for gastritis throughout the intervention.

### 2.4. Tactile Massage Intervention

Tactile massage treatments were conducted in two cities within centrally located premises linked to well‐being to ensure a neutral massage environment. A total of 129 tactile massage sessions were delivered by seven female massage therapists, all certified in the TaktiPro method [22]. Certification as a TaktiPro therapist includes 2 days of theoretical and practical training, followed by 60 h of supervised hands‐on practice and successful completion of both theoretical and practical examinations. The massage therapists recruited for this study were external to the Child and Adolescent Clinic and represented a range of professional backgrounds.

The adolescents received 10 weekly sessions of tactile massage following a standardised protocol. Each 60‐min session used structured, gentle strokes applied in a slow, calm rhythm to stimulate skin‐level tactile receptors. The massage followed a fixed sequence (back, hands, feet and a final full‐body stroke) to ensure consistency and familiarity. Sessions were conducted with neutral oil, soft music and full‐body coverage, exposing only the area being massaged. For further information, see Robertz et al., 2026 [23].

### 2.5. Data Collection

#### 2.5.1. Hair Sampling

All hair samples were collected by A‐C‐R and one research assistant between January and September 2022. For each participant, hair was consistently cut in lengths measuring between 3–9 cm from the same area closest to the scalp. Our analysis and report are based on the results of hair cortisol closest to the scalp from hair samples measuring 3 cm in length. The weight of each hair sample was 5 mg or more. The samples were placed in plastic tubes wrapped in cellophane and stored at room temperature in a dark, fireproof box until analysis to minimise the risk of reduced hair cortisol concentration due to ultraviolet exposure [24].

#### 2.5.2. Cortisol Analysis

To extract and analyse cortisol concentrations in hair we used a competitive radioimmunoassay (RIA), which enables the analysis of small samples [5]. This method has previously been described by Karlen and Morelius—for details, see File S1 [5, 25]. The samples were double samples, analysed at the same time point.

#### 2.5.3. Heart Rate and Blood Pressure Measurement

Blood pressure and heart rate were measured before the intervention at T0, during the intervention after each massage session at T1 to T3, and 3 months after the intervention at T4. Blood pressure was measured using a fully automated electronic upper‐arm blood pressure monitor (Omron M3 Comfort 2020, HEM‐7155‐E; manufactured in 2021). Appropriate cuff size was determined by measuring upper‐arm circumference at baseline (T0). Heart rate was recorded automatically by the device. The first time blood pressure was taken, measurements were recorded for both upper arms to control for side differences. Three consecutive measurements were obtained, with mean values calculated in accordance with standardised clinical guidelines [26]. Thereafter, blood pressure was consistently taken on the arm with the higher value and performed as a single measurement, which was documented and used across all measurement time points (T1–T4). These measurements were used in all calculations.

#### 2.5.4. SIC

The SIC questionnaire [21] assesses perceived stress using 21 items rated on a 4‐point Likert scale from ‘never’ to ‘very often’, including 13 positively phrased items to reduce bias. A total score is obtained by adding up all the items, with higher scores indicating greater perceived stress. The entire SIC questionnaire has a Cronbach′s alpha of 0.86. The SIC questionnaire was answered at T0 to T4 to estimate the effect of tactile massage on stress and compare with hair cortisol concentration at the same time points. See File S2, SIC questionnaire Version 2009.

### 2.6. Ethical Considerations

The study was conducted in accordance with the Declaration of Helsinki and good clinical practice (GCP). Ethical approval was obtained from the Swedish Ethical Review Authority (Dnr. 2020‐01414).

### 2.7. Statistical Analysis

The statistical analysis was performed with figures created in the software programmes STATISTCA Statistics for Windows, Version 14.2.0.18, 1984–2023 Cloud Software Group, Germany, (2024). To assess the symmetry of the results, a histogram was created, which showed a non‐symmetric distribution. Consequently, nonparametric statistical methods were applied. Data were log 10‐transformed before statistical calculations. Descriptive data were used to give background data and hair cortisol concentrations at different time points. For descriptive purposes, hair cortisol is provided in pg/mg and in median (Mdn) and interquartile range (IQR) in Table 2. When the expected cell count was five or fewer in Tables 1 and 2, Fisher′s exact test was used to compare the variables presented in the tables. To compare groups, we used Mann–Whitney *U*, Median test and Kruskal–Wallis ANOVA by ranks tests. To look for trends, we used Friedman′s ANOVA. For analysis of time‐dependent changes within a participant, we used Wilcoxon′s matched‐pairs test. Spearman′s correlation test was used for the estimation of correlations. *p* − level < 0.05 was considered statistically significant.

**Table 2:** Hair cortisol concentration in children before, during and at follow‐up of tactile massage 3 months postintervention.

Cortisol (pg/mg)	T0	T1	T2	T3	T4
Female	[5] 12.8 (2.3)	[5] 16.2 (22.1)	[5] 12.7 (1.1)^a^	[5] 13.9 (3.3)	[5] 12.6 (3.8)
Male	[9] 16.2 (8.1)	[9] 17.1 (7.4)	[8] 14.2 (7.3)^a^	[8] 16.3 (8.8)	[6] 16.0 (6.9)
All	[14] 13.6 (8.5)	[14] 16.7 (11.6)	[13] 13.5 (1.5)	[13] 14.1 (5.9)	[11] 14.6 (7.9)

*Note:* T0 (at baseline), T1 (immediately before first tactile massage), T2 (at sixth tactile massage), T3 (at 10th tactile massage) and T4 (12 weeks after last tactile massage). Values are given as (*n*), median and interquartile range (IQR).

^a^There was a significant difference in hair cortisol concentration after six tactile massages when comparing for sex, *p* = 0.029.

## 3. Results

### 3.1. Time Points for Sampling and Analysis of Hair Cortisol Concentration

Samples represent the long‐term cortisol levels from Autumn 2021 to September 2022. They were analysed simultaneously in April 2023. The limit range for the RIA analysis was 2–3000 pg/mg. Hair cortisol concentration varied widely between individual participants, from 7–637 pg/mg. The two highest values occurred in a 16‐year‐old girl (participant L) before tactile massage. There were no significant differences in hair cortisol levels among participants within the 15–17‐year age range (Mann–Whitney *U*, *n* = 59; *p* = 0.64).

Hair cortisol concentration (log10‐transformed) for all 14 participants from January to September 2022 is illustrated in Figure 2.

**Figure 2:**
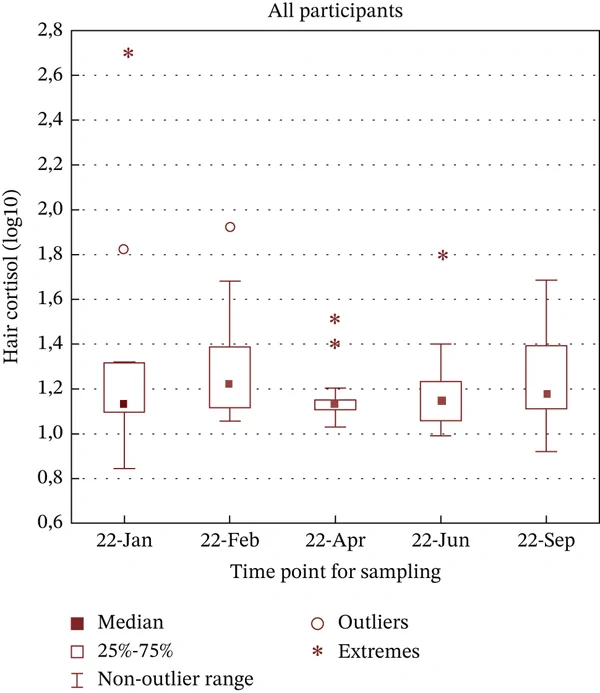
Hair cortisol concentration (log 10 transformed) for all 14 participants from January to September 2022.

Table 2 shows long‐term cortisol in the 3 months preceding the intervention. Hair cortisol concentrations were not significantly different from baseline when comparing participants as a whole (Friedman ANOVA, *p* = 0.64) or when split by sex (Wilcoxon matched‐pairs test, *p* > 0.05). However, a significant difference in hair cortisol concentration was seen after six tactile massage sessions when comparing sex (Mann–Whitney *U*, *p* = 0.029).

We found no significant correlations at T0 between hair cortisol concentration and body weight (*n* = 14, rho = −0.015; *p* = 0.96), height (*n* = 14, rho = 0.17; *p* = 0.57) and body mass index (BMI) (*n* = 13, rho = −0.35; *p* = 0.25) (Spearman′s correlation).

### 3.2. Effects of Tactile Massage on Hair Cortisol Concentration

Figure 3 illustrates the progression of hair cortisol concentration before, during and after tactile massage. There was no significant difference in hair cortisol concentration between time points (T0 to T4) when analysing all adolescents (Kruskal–Wallis ANOVA, *p* = 0.67) or split by sex (Wilcoxon matched‐pairs test, *p* > 0.05).

**Figure 3:**
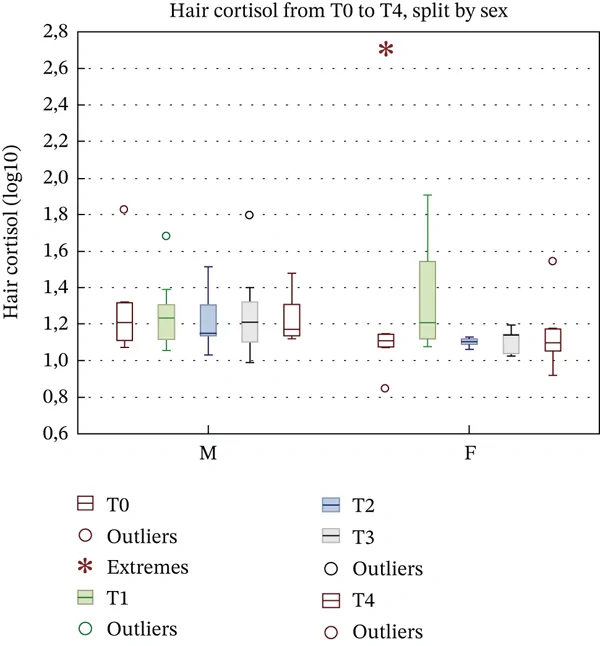
Hair cortisol concentration before (T0 + T1), during (T2 + T3) and at follow‐up of tactile massage (T4) for all participants, split by sex. Note: T0 (at baseline), T1 (immediately before first tactile massage), T2 (at sixth tactile massage), T3 (at 10th tactile massage). M = male, F = female. Values are given as median, interquartile range, outliers and extremes.

When comparing time periods before (T0 + T1), during (T2 + T3), and after tactile massage (T4), there was no significant difference according to the Kruskal–Wallis ANOVA (*p* = 0.78) and Median test (*p* = 0.69), respectively. However, when comparing hair cortisol concentration after six tactile massages split by sex (M = male, F = female), females showed significantly lower hair cortisol concentration (Mann–Whitney *U*, *p* = 0.029) than males (see Table 2).

For a more detailed comparison of time periods before and during tactile massage, we compared the mean hair cortisol concentration of the first (T0) and second (T1) samples (before tactile massage) with the mean hair cortisol concentration of the third (T2) and fourth samples (T3) (during tactile massage) (Wilcoxon matched‐pairs test). Even then, no significant difference in hair cortisol concentration was found when considering the participants as a whole or split by sex (see Figure 4).

**Figure 4:**
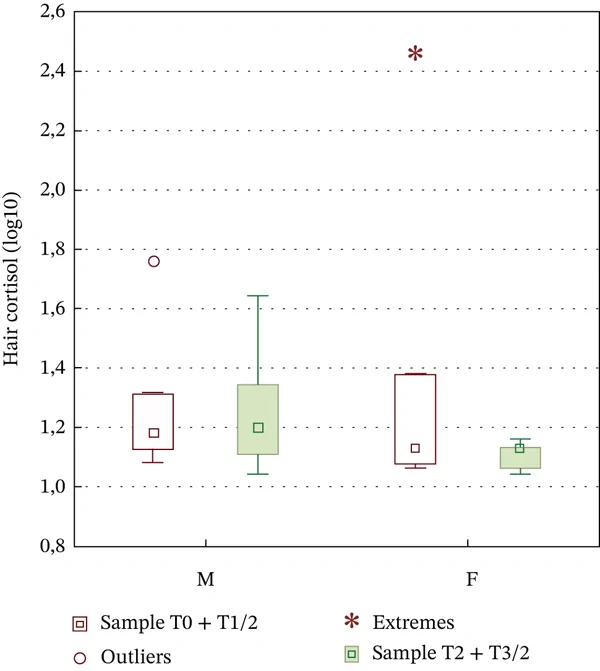
Hair cortisol concentration before (T0 + T1) and during (T2 + T3) tactile massage, split by sex. Note: T0 (at baseline), T1 (immediately before first tactile massage), T2 (at sixth tactile massage), T3 (at 10th tactile massage). M = male, F = female. Values are given as median, interquartile range, outliers and extremes.

The individual pattern of hair cortisol concentration during the whole study period is illustrated in Figure 5. Two individuals (participants K and L) had very high hair cortisol concentration at baseline (concentration > 60 pg/mg) followed by a clear decrease approaching the hair cortisol concentration levels of other participants at ≤ 20 pg/mg. No known deviations or other circumstances that could explain the higher concentrations in these two participants were identified. Between T3 and T4, only two individuals (participants J and H) showed an increase in hair cortisol concentration (Figure 5). Notably, participant J initiated SSRI treatment at T3 following completion of the TM intervention, and the increase in hair cortisol was observed at the three‐month follow‐up (T4).

**Figure 5:**
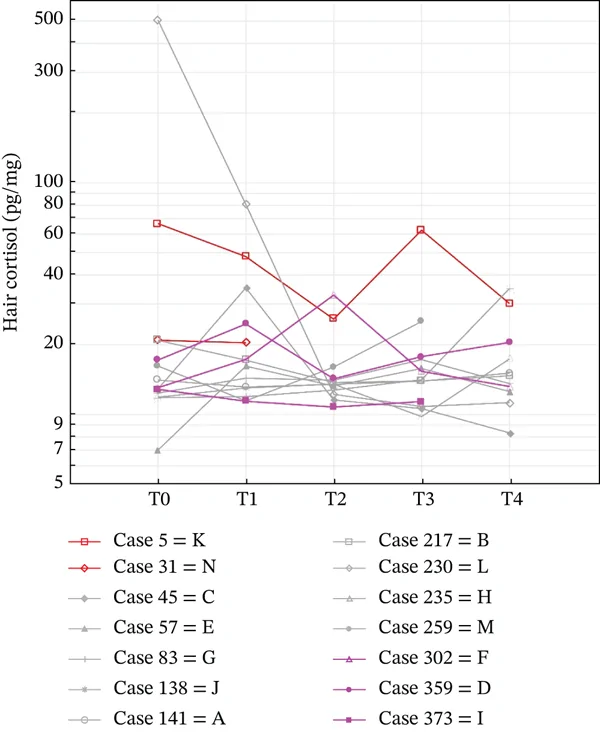
Individual hair cortisol concentrations before, during and after tactile massage. Note: Hair cortisol concentration is on a logarithmic scale. T0 (2 months before first tactile massage), T1 (immediately before first tactile massage), T2 (after sixth tactile massage), T3 (after 10th tactile massage), T4 (3 months after intervention). Case numbers are from the randomisation process. The 14 individuals are labelled A–N for comparison of hair cortisol concentrations with other outcomes in previous publication [20].

### 3.3. Heart Rate and Blood Pressure

Five participants had increased systolic blood pressure (> 120 mmHg), four had increased diastolic blood pressure (> 80 mmHg) and three participants had a heart rate of over 100 beats per minute (bpm) when controlled at each tactile massage session. These participants did not have abnormally high hair cortisol concentrations. There were no correlations between hair cortisol concentration and heart rate (*n* = 65, rho = 0.09, *p* = 0.457), diastolic blood pressure (*n* = 65, rho = −0.06, *p* = 0.627) and systolic blood pressure (*n* = 65, rho = 0.10, *p* = 0.435) (Spearman′s correlation).

### 3.4. Hair Cortisol in Relation to Stress Estimated in SIC Questionnaire

Hair cortisol concentration was compared with the SIC questionnaire scores before tactile massage in January 2022 and with the last time point in September 2022 at follow‐up. The grade of stress, estimated from total scores for SIC, was compared with hair cortisol concentration at the same time point. The three subscales, well‐being, frustration and social support, were also estimated and compared to hair cortisol concentration. When all hair cortisol concentration samples (*n* = 64) were compared with total SIC scores, there was no correlation (Spearman′s correlation) (rho = −0.19, *p* = 0.126). However, there was a significant negative correlation between hair cortisol concentration and the subscale SIC frustration (rho = −0.27, *p* = 0.028), but not for the subscales well‐being (rho = −0.02, *p* = 0.849) and social support (rho = −0.04, *p* = 0.767), not shown.

## 4. Discussion

This study examined long‐term HPA axis activity, measured through hair cortisol concentrations in adolescents with ADHD before, during and after a structured tactile‐massage intervention. By integrating repeated biological sampling with physiological and self‐reported stress measures, it offers a comprehensive assessment of long‐term stress‐system regulation in a well‐characterised adolescent ADHD cohort.

### 4.1. Hair Cortisol as a Marker of Long‐Term HPA‐Axis Activity in ADHD

Hair cortisol concentration has been recognised as a reliable biomarker of cumulative cortisol secretion over weeks to months, reflecting long‐term HPA‐axis activity rather than acute or diurnal fluctuations [5, 27]. In the present study, hair cortisol concentration showed substantial interindividual variability but high intraindividual stability across the observation period, spanning up to 1 year. The wide range of baseline hair cortisol concentration values observed aligns with earlier findings in both clinical and community‐based samples of children and adolescents, including those with psychological distress or neurodevelopmental disorders [15, 16, 28].

### 4.2. ADHD, Cortisol Regulation and Heterogeneity of Findings

Research on cortisol regulation in ADHD has yielded heterogeneous findings. Meta‐analytic evidence indicates that children and adolescents with ADHD, particularly those recruited from clinical settings, tend to exhibit lower basal cortisol levels and reduced cumulative cortisol output compared with typically developing controls [3]. These findings have been interpreted as potentially linked to altered arousal regulation, reduced stress sensitivity or adaptive responses to chronic stress exposure. However, systematic reviews and empirical studies emphasise that cortisol alterations in ADHD are not uniform and are strongly influenced by psychiatric comorbidity [2, 4]. Blunted cortisol responses and lower basal cortisol levels are most consistently observed in individuals with ADHD when comorbid externalising disorders are present [4]. Recent adolescent data further indicate that when comorbid anxiety, depression and externalising symptoms are statistically controlled, associations between ADHD symptoms and cortisol output may shift direction or attenuate [6]. The cohort in the present study was homogeneous, which may partly explain the absence of great systematic changes in hair cortisol concentration at baseline and until the last session.

### 4.3. Effects of Tactile Massage on Long‐Term Cortisol Secretion

The primary intervention‐related finding was the absence of a consistent, sustained reduction in hair cortisol concentration following tactile massage at the group level. The observed changes in our study were not uniform, and no overall long‐term effect was detected at follow‐up. Consequently, neither the hypothesis that tactile massage would induce a sustained reduction in long‐term cortisol secretion nor the hypothesis that hair cortisol would be sufficiently sensitive to detect such intervention‐related changes was supported. However, if identical tactile massage had been given more frequently, for instance twice weekly, or weekly for a longer period, the results may have been different. It is important, therefore, to investigate this in future studies of tactile massage. The findings contrast with earlier studies reporting reductions in salivary cortisol directly following tactile massage or related interventions [29], but such discrepancies are likely attributable to fundamental differences between acute and cumulative cortisol measures. For future studies, it would seem optimal to combine both salivary and hair cortisol analysis. Importantly, the lack of a long‐term hair‐cortisol effect does not rule out potential clinical or psychological benefits. In our earlier work with the same participants, self‐report measures indicated reductions in hyperactivity and improvements in attention following tactile massage [20].

### 4.4. Sex Differences and Developmental Considerations

A notable finding was the sex difference in hair cortisol concentration observed after six tactile massage sessions, with boys exhibiting higher hair cortisol concentration than girls. Sex differences in hair cortisol concentration during childhood and adolescence have been inconsistently reported in the literature. Some studies have found higher hair cortisol concentration in boys, others higher levels in girls and several report no sex differences at all [8, 15, 27, 30]. Developmental stage, pubertal status and psychological distress appear to moderate these associations. ADHD‐specific research further supports sex‐dependent and phenotype‐dependent cortisol patterns. Pauli‐Pott et al. [2] demonstrated that hair cortisol concentration varied according to ADHD symptom presentation, comorbidity and sex, with low hair cortisol concentration particularly associated with inattentive symptoms in boys. Llorens et al. [1] similarly found that higher hair cortisol concentration was associated with better attention and memory performance in boys with ADHD, suggesting that cortisol may index adaptive arousal or cognitive engagement in certain subgroups. The sex difference observed in the present study may therefore reflect differential stress regulation mechanisms in male and female adolescents with ADHD rather than a direct effect of the intervention.

### 4.5. Hair Cortisol Concentration in Relation to Autonomic Measures and Perceived Stress

No associations were observed between hair cortisol concentration and heart rate or blood pressure across the study period. These findings align with previous research indicating that HPA‐axis activity and sympathetic nervous system responses represent partially independent stress‐regulatory systems [31, 32].

Total perceived stress, as measured by the SIC questionnaire, was not correlated with hair cortisol concentration. This dissociation between subjective stress and biological stress markers is well documented and underscores the complexity of stress regulation [5, 33]. Interestingly, a negative association was observed between hair cortisol concentration and the SIC frustration subscale, suggesting that adolescents with higher long‐term cortisol output reported lower frustration. Comparable findings have been reported in studies linking hair cortisol concentration to resilience, optimism and coping styles rather than to global stress ratings [33]. These results support the interpretation that hair cortisol concentration may reflect broader regulatory capacity or adaptive engagement rather than perceived stress alone.

### 4.6. Effect of Pharmacotherapy on Cortisol Concentration

Participant J, who initiated SSRI treatment at T3, showed an increase in hair cortisol between T3 and T4. This is consistent with a study that reported increased hair cortisol following 4 weeks of antidepressant treatment in individuals with depression, whereas levels remained unchanged in healthy controls. Although this temporal association is noteworthy, it should be interpreted cautiously given the single‐participant observation and the potential influence of other factors. For participant H, who also showed increased hair cortisol levels between T3 and T4, no apparent explanation for this increase was identified [34].

## 5. Strengths and Limitations

The methodological strengths of this study include the repeated‐measures design, the extended follow‐up period and the use of a well‐validated RIA for hair cortisol analysis with low intra‐assay and interassay variability [5]. Nevertheless, several limitations warrant consideration. The sample size was small, limiting statistical power and the ability to detect subtle subgroup effects. The absence of a control group precludes direct comparison of hair cortisol concentration levels between adolescents with ADHD and typically developing peers. Two participants did not receive the full intervention dose, although their inclusion did not materially alter the results.

## 6. Conclusions

Our findings suggest that hair cortisol concentration has limited sensitivity for detecting intervention‐related effects of tactile massage in this population over the studied time frame. The absence of clear changes in hair cortisol concentration following the intervention likely reflects the trait‐like nature of long‐term cortisol secretion and the complexity of stress regulation in ADHD, although methodological factors, such as the relatively small sample size, cannot be ruled out. Our findings suggest that hair cortisol concentration may be a relatively coarse measure for capturing meaningful intervention‐related changes in adolescents with ADHD and may therefore have limited utility as a primary outcome measure in this context.

Future research should include larger sample sizes, healthy control groups and multiple methods of assessing stress, such as hair cortisol concentration, salivary cortisol, autonomic indicators and real‐time self‐reports collected during daily life.

**Nomenclature**
ADHD: attention‐deficit/hyperactivity disorder
BMI: body mass index
BP: blood pressure
Bpm: beats per minute
F: female
GCP: Good Clinical Practice
HPA‐axis: hypothalamic–pituitary–adrenal axis
ICD‐10: International Classification of Diseases Version 10
IQR: interquartile range
M: male
Mdn: median
MINI‐KID: Mini‐International Neuropsychiatric Interview for Children and Adolescents
ODD: oppositional defiant disorder
RIA: radioimmunoassay
SD: standard deviation
SIC: Stress in Children questionnaire
SSRI: selective serotonin reuptake inhibitor
T0: at baseline
T1: immediately before first tactile massage
T2: at sixth tactile massage
T3: at 10th tactile massage
T4: 12 weeks after last tactile massage

## Author Contributions

Carl‐Johan Törnhage and Anna‐Carin Robertz drafted the initial manuscript and were responsible for adapting the draft to comply with the author guidelines. Carl‐Johan Törnhage was responsible for the statistical analysis, tables and illustrations. Anna‐Carin Robertz was responsible for hair sampling, cardiovascular measurements, the questionnaire and illustrations. Anne‐Katrin Kantzer critically reviewed and revised the manuscript. Stefan Nilsson critically reviewed and revised the manuscript and supervised the study. All authors contributed to the conceptualisation and study design, data extraction, writing and revision of the manuscript. Carl‐Johan Törnhage and Anna‐Carin Robertz contributed equally to the manuscript.

## Funding

This study was funded by the Ekhagastiftelsen, 10.13039/501100021592, 2020‐16; Bengt och Elna Molin; Partikulare; SSSH (Södra Sveriges Sjuksköterskehem); Peter Silfverskiöld, 2025‐670; Hvitfeldska; Ebba Danelius; Majblommans Riksförbund, 10.13039/501100009768, 27; FRF (Fonden för Rehabilitering och Medicinsk Forskning); Department of Research and Development, NU‐Hospital Group; and the Fyrbodal Research and Development Council Region Västra Götaland.

## Disclosure

All authors approved the final manuscript as submitted and agreed to be accountable for all aspects of the work.

## Conflicts of Interest

The authors declare no conflicts of interest.

## Supporting Information

Additional supporting information can be found online in the Supporting Information section.

## Supporting information


**Supporting Information 1** File S1: Hair cortisol analysis method—a cortisol measurement using a competitive radioimmunoassay in methanol extracts of hair samples, frozen in liquid nitrogen and mechanically pulverised. Hair samples of 5 mg or more were needed.

**Supporting Information 2** File S2: Stress in Children (SIC)—a questionnaire including 21 items on physical health issues (headache, abdominal pain and sleep) and mental health and rated on a four‐grade Likert scale (*never*, *sometimes*, *often* and *very often*). The child provides the answers and parents or teachers can explain the questions to some degree but with no other comments or recommendations.

## Data Availability

Data are available upon request from the corresponding author.
