# Supplementary material for: Methodological Considerations for Using Hair Cortisol as a Biomarker Following No Measurable Effects of Tactile Massage in Adolescents With ADHD
Source: Int J Pediatr. 2026 Oct 21;2026:5891199. doi: 10.1155/ijpe/5891199 (PMC13685493; doi:10.1155/ijpe/5891199)
Supplement: Supplementary file 1 [file IJPE-2026-5891199-s001.docx]

Supplementary File2	**Stress in Children questionnaire**

**Frågeformulär**

Detta frågeformulär handlar om stress i skolan. Svaren skall utan påverkan fyllas i av barnet själv. Frågan får dock förklaras av lärare eller förälder. Ifyllandet sker individuellt och utan påverkan från andra.

**Jag heter** ______________________________________ **Hur gammal är du?_______**

**Besvara frågorna här nere så gott du kan. Försök att svara på alla frågorna.**

**Först läser du frågan, sen sätter du ett kryss i den ruta som passar bäst med hur du har det.**

**Aldrig	Ibland	Ofta	Mycket	ofta**

**1. Jag blir arg	❑	❑	❑	❑**

**2. Jag har huvudvärk	❑	❑	❑	❑**

**3. Jag tycker det är roligt**

**att gå till skolan	❑	❑	❑	❑**

**4. Jag känner mig lugn och glad	❑	❑	❑	❑**

**5. Jag har ont i magen	❑	❑	❑	❑**

**6. Jag känner mig ensam	❑	❑	❑	❑**

**7. Jag blir ledsen	❑	❑	❑	❑**

**8. Jag tycker om att vara i**

**skolan	❑	❑	❑	❑**

**9. Kamraterna retar mig	❑	❑	❑	❑**

**Aldrig	Ibland	Ofta	Mycket	ofta**

**10. Jag har lätt att somna på**

**kvällarna	❑	❑	❑	❑**

**11. Jag känner mig lugn	❑	❑	❑	❑**

**12. Det brukar bli som jag**

**tänkt mig	❑	❑	❑	❑**

**13. Jag känner mig glad	❑	❑	❑	❑**

**14. När jag är glad visar jag det	❑	❑	❑	❑**

**15. Det händer att jag inte hinner**

**med det jag ska	❑	❑	❑	❑**

**16. När det är jobbigt hjälper det**

**att vara med kamraterna	❑	❑	❑	❑**

**17. När jag är ledsen visar jag det	❑	❑	❑	❑**

**18. Det händer att jag inte klarar**

**av det jag ska	❑	❑	❑	❑**

**19. När det är jobbigt finns det**

**någon vuxen jag kan prata med	❑	❑	❑	❑**

**20. När någon är dum mot mig**

**så säger jag ifrån	❑	❑	❑	❑**

**21. Det går lätt att koncentrera**

**sig på lektionerna	❑	❑	❑	❑**
