# Supplementary material for: Methodological Considerations for Using Hair Cortisol as a Biomarker Following No Measurable Effects of Tactile Massage in Adolescents With ADHD
Source: Int J Pediatr. 2026 Oct 21;2026:5891199. doi: 10.1155/ijpe/5891199 (PMC13685493; doi:10.1155/ijpe/5891199)
Supplement: Supplementary file 2 [file IJPE-2026-5891199-s002.docx]

**Supplementary file 1** Haircortisol analyse method

The hair samples were cut into smaller pieces, frozen for 2 min in liquid nitrogen, and minced together with a 5 mm steel ball using a Cry Mill for 10 min. Methanol (1ml) was added to each tube, and the samples were extracted overnight on a moving board. Then, 0.8 mL of the methanol supernatant was pipetted off and lyophilised using a Savant Speed Vac Plus SC210A. The lyophilised extracts of hair samples were dissolved in 300 μL 0.1 mmol/lL phosphate buffer, pH 7.4, containing 0.02% bovine serum albumin and a 0.01% triton Z-100, and cortisol concentrations were analysed as described by (23) Morelius et al. (2004). Hair samples of 5 mg or more were needed to maintain a total inter-assay coefficient of variation below 8% for hair extraction and measurement of cortisol by the RIA. The intra-assay coefficient of variation for the RIA itself was 7% at 10 nmol/L (5). Furthermore, the gamma radiation used for detection in the RIA method is not influenced by coloured hair, which is extracted with methanol together with the cortisol.
